# How is loneliness orientation implicated in the relationship between sleep problems, loneliness intensity, and school refusal in adolescents?

**DOI:** 10.1007/s41105-025-00586-9

**Published:** 2025-04-24

**Authors:** Isa Okajima, Setoka Hiruma

**Affiliations:** 1 Behavioral Sleep Medicine and Sciences Laboratory, Department of Psychological Counseling, Faculty of Humanities, Tokyo Kasei University, 1-18-1 Kaga, Itabashi-ku, Tokyo 173-8602 Japan; 2 Maebashi City Tsukida Elementary School, 273 Kasukawamachi Tsukida, Maebashi, Gunma 371-0203 Japan

**Keywords:** Loneliness, Orientation, Insomnia, Sleep debt, Chronotype, School refusal

## Abstract

Prolonged loneliness adversely affects mental and physical health. Even if feelings of loneliness are high, the association between loneliness intensity, mental health, and school refusal may differ depending on how loneliness is framed (i.e., loneliness orientation). In this study, we aimed to develop a Loneliness Orientation Test to examine the relationship between loneliness intensity and loneliness orientation; examine the differential impact of sleep problems on loneliness intensity/orientation, feelings of school refusal, and the number of absences; and examine the relationship between the combined types of loneliness intensity with loneliness orientation, sleep problems, and school refusal. The study sample included 168 senior high school students. We used loneliness-related measures, including the Loneliness Oriented Test developed in this study, sleep-related measures (insomnia, sleep debt, and chronotype), and school refusal-related measures (feelings of school avoidance and absenteeism). These findings showed that feelings of school avoidance and absenteeism were associated with different sleep problems (insomnia, sleep debt, and chronotype). Additionally, one-fourth of lonely individuals viewed loneliness positively; those with a positive loneliness orientation did not have worse sleep conditions than those with a negative loneliness orientation. Even when loneliness intensity is strong, outcomes such as sleep problems vary depending on an individual's orientation toward loneliness.

**Supplementary Information:**

The online version contains supplementary material available at 10.1007/s41105-025-00586-9.

## Introduction

Loneliness has recently become a global concern. In the USA, it has been reported that approximately 50% of people feel lonely sometimes or always [1], while in the UK, 68% of people feel lonely sometimes, always, or often [2]. Loneliness is a feeling encountered throughout the lifespan, yet the reasons for loneliness vary with age [3]. In particular, young adults aged 18–22 are most likely to feel lonely [4].

### Negative aspects of loneliness

Previous studies have investigated the negative impact of loneliness intensity on alcohol use [5], depression [6], suicide risk [7], and mortality risk [8]. During the school years, loneliness intensity is related to sleep problems [9], poor academic outcomes [10], and school refusal [11]. Sleep issues can lead to increased feelings of school refusal both directly and indirectly, with loneliness serving as a mediating factor in this relationship [9]. In Japan, when a student misses 30 or more days of school per year—not counting absences due to illness or financial hardship—this is categorized as school refusal. Therefore, we must differentiate between these contributing factors and the actual duration of school absences. Furthermore, although it has been suggested that increased feelings of school refusal can lead to an increased number of absences [12], the link between feelings of school refusal and the number of absences has not been clarified. Nearly 99% of Japanese students proceed to senior high school [13], and graduation from senior high school has become the fundamental prerequisite for young Japanese individuals seeking employment [14]. Consequently, school refusal in senior high school may impede employment opportunities and social autonomy, perhaps resulting in heightened feelings of loneliness in young adulthood. Furthermore, therapies aimed at diminishing loneliness intensity—such as meditation/mindfulness and social support—have been recently conducted; nevertheless, the strength of the evidence is very low, and their effectiveness is not stable [15].

### Positive aspects of loneliness

Conversely, it seems that loneliness itself is not problematic at any age any more than hunger, thirst, or pain from an evolutionary perspective [3]. The reaffiliation motive (RAM) model has been suggested as a positive aspect of overcoming loneliness. A RAM consists of three component processes that promote reconnection [3]: The aversive feeling of loneliness, the awareness that one is lonely, and an increase in implicit vigilance for social threats. When the RAM works well, they promote the development of salutary relationships with others. According to this model, loneliness feelings are often a generally transient experience but sometimes lead to prolonged loneliness with negative cognition and feelings about the world. Prolonged loneliness can adversely affect mental and physical health. On the other hand, therefore, it is not well clarified which individuals suffer from malfunction of RAM and toward prolonged loneliness.

One study reported that among individuals with high-intensity loneliness feelings, those with a high positive affect (e.g., “excited,” “interested,” and “active”) had a lower mortality risk than those with a low positive affect [8]. That is, even if people who have high feelings of loneliness, those with positive aspects of overcoming loneliness may differ the association between loneliness, mental health, and school refusal than they with negative aspects of loneliness; therefore, it is important how loneliness is framed (i.e., loneliness orientation). However, no studies on this perspective have been conducted so far.

In this study, we aimed to develop a Loneliness Orientation Test (LOT) to evaluate loneliness orientation and to examine: the relationship between feelings of loneliness and loneliness orientation; the differential impact of sleep problems on loneliness, feelings of school refusal, and the number of absences; and the relationship between the combined types of loneliness intensity with loneliness orientation, sleep problems, and school refusal. Clarifying the characteristics of combined loneliness intensity and loneliness orientation may lead to the development of approaches to reduce loneliness.

## Methods

The Ethics Committee at Tokyo Kasei University approved this research project (approval number: Ita-E2023-16). Following the guidelines set forth in the Declaration of Helsinki, researchers obtained informed consent from all participants before their involvement in the study.

### Participants

The data analyzed in the in-person survey was collected from November to December 2023. The sample comprised 168 students from public and private senior high schools in Tokyo. Of these students, 128 (52 males, 70 females; mean age: 16.55 ± 0.64 years) who completed the following measures and who had a sleep debt index (SDI) score of ≥0, based on a previous study [16], were selected and analyzed.

### Measures

#### Loneliness-related measures

*Loneliness Orientation Test (LOT)*. To evaluate loneliness orientation, we developed the LOT using a semantic differential method. The scale asked for responses to each of seven pairs of words (Table 1) regarding their image of “loneliness,” using a seven-item method (−3 to 3). Negative and positive scores indicated negative and positive orientations towards loneliness, respectively.

**Table 1:** Result of an EFA and CFA of the LOT

Items	EFA		CFA
	Factor loadings	Communality	Factor loadings
Item 5: Decline—Growth	0.85	0.73	0.85
Item 4: Negative—Positive	0.82	0.68	0.82
Item 2: Retroactive—Proactive	0.82	0.67	0.82
Item 7: Unhappy—Happy	0.79	0.63	0.79
Item 6: Avoidance—Approach	0.79	0.62	0.79
Item 3: Collapse—Overcome	0.78	0.61	0.78
Item 1: Bad—Good	0.76	0.58	0.76
Factor contribution	4.51	–	–
Proportion of factor contribution	0.64	–	–

*CFA* Confirmatory factor analysis, *EFA* Exploratory factor analysis, *LOT* Loneliness Orientation Test

*Three-Item Loneliness Scale (TILS)*. The scale is a validated self-report tool that measures loneliness intensity through three questions [17, 18]. A higher total score indicates greater feelings of loneliness.

#### Sleep-related measures

*Athens Insomnia Scale (AIS).* This eight-item self-report questionnaire evaluates insomnia severity [19–21]. Total scores are calculated by adding individual responses, with higher scores reflecting more severe insomnia symptoms. Participants scoring 6 or above were classified as having clinical insomnia, based on the established cutoff point of 5.5.

*SDI.* The scale is calculated from responses to three questions about sleep duration: (1) sleep duration on weekdays, (2) sleep duration on weekends, and (3) preferred sleep duration when free from obligations. Following Okajima et al.’s methodology [16], the SDI calculation produces higher scores for greater sleep debt.

*Circadian Energy Scale (CIRENS).* This validated two-item scale measures chronotype (morning vs. evening preference) using energy ratings from 1 (very low) to 5 (very high) [22]. The final score is calculated by subtracting morning energy from evening energy, resulting in a range from -4 (strong morning preference) to +4 (strong evening preference).

#### School-refusal-related measures

*Feeling of School-Avoidance Scale (FSAS).* The scale measures attitudes toward school attendance [23]. This tool includes six items that assess aversion to school, such as “I want to miss school” and “I want to go home as soon as classes are over.” Higher scores indicate stronger resistance to school attendance.

*Number of absences.* Participants were asked to provide information on the number of days absent between April 2023 and the time of the survey.

### Sample size

The sample size was calculated using a power analysis that examined the correlation coefficients (*r*) among AIS, TILS, and FSAS in Okajima's study [9]. Previous findings showed significant correlations (*p*<0.01) between all three measures: AIS–TILS (*r*=0.33), AIS–FSAS (*r*=0.43), and TILS–FSAS (*r*=0.49). Using a statistical power of 0.8, the analysis indicated that between 44 and 102 participants would be needed. To ensure adequate sampling, the researchers aimed to recruit at least 110 participants.

### Statistical analysis

Descriptive statistics were computed using the R statistical software (version 4.4.0; R Project for Statistical Computing, Vienna, Austria). We utilized the R packages as follows: “pwr,” “psych,” for descriptive statistics, “GPArotation,” “lavaan,” for factor analysis, “NbClust,” “factoextra,” “Rmisc” for cluster analysis, “tidyyverse,” “MASS,” “dplyr,” “easystats,” “ggeffects,” “lme4” “effectsize” for generalized linear models (GLM), and “compute.es.” for Hedges’ g.

First, the structural validity of the LOT was evaluated using exploratory factor analysis (EFA) with a maximum likelihood solution method. The factors were determined based on the shape of the scree plot using parallel analysis. In addition, we estimate a goodness of fit of the factor structure using confirmatory factor analysis (CFA).

Second, correlation analysis was conducted between the scales. To confirm the association between sleep problems, loneliness, and school refusal, we conducted a regression analysis using a GLM with (1) sleep problems (AIS, SDI, and CIRENS) and the LOT as independent variables and the TILS as the dependent variable using the Gamma distribution with a log-link function; (2) sleep problems, the LOT, and TILS as independent variables, and school refusal as dependent variables using the Gamma distribution with a log-link function; and (3) sleep problems, the LOT, and TILS as independent variables, and the number of absences as dependent variables using the Poisson distribution with a log-link function.

Third, we performed a hierarchical cluster analysis (CA) with Ward method to investigate the combination of feelings of loneliness (TILS) and loneliness orientation (LOT). We determined the best number of clusters based on the results of some methods such as silhouette and gap statistic. We then conducted an analysis of variance using a GLM. Multiple comparisons were performed using Dunnett’s method when the estimated values were significantly different. We estimated the effect sizes of scales between the cluster groups using Hedges’ g. In general, an absolute g value of 0.2 indicates a small effect size, a value of 0.5 indicates a moderate effect size and a value of 0.8 indicates a large effect size [24].

For some parameters, 95% confidence intervals (CIs) were used; if the CI did not equal zero, it was considered statistically significant.

## Results

### Factor structure of the LOT

The results of EFA with parallel analysis confirmed a one-factor structure (Table 1). Cronbach’s α was 0.94. We performed CFA based on the result of EFA. The result was showed that a one-factor structure model was a good fit (χ^2^_14_=16.58, *p*=0.28, CFI=0.99, RMSEA=0.05), and McDonald’s *ω* was 0.94. The total score divided by the number of items was used as the LOT score.

Correlation analysis revealed that the CIRENS (*r*=0.33), SDI (*r*=0.37), FSAS (*r*=0.37), and TILS (*r*=0.29) were significantly associated with the AIS and CIRENS with SDI (*r*=0.24; Table 2). The FSAS correlated with TILS (*r*=0.52) and the number of absences (*r*=0.24), and the LOT negatively correlated with SDI (*r*=−0.12; Table 2).

**Table 2:** Correlation coefficients between scales

	AIS	SDI	CIRENS	TILS	LOT	FSAS	No. of Absences
AIS	–	**0.37**	**0.33**	**0.29**	−0.07	**0.37**	0.15
SDI	**[0.21, 0.51]**	–	**0.24**	−0.04	−**0.12**	0.07	−0.03
CIRENS	**[0.17, 0.48]**	**0.07, 0.40**	–	−0.02	−0.16	0.06	0.11
TILS	**[0.13, 0.44]**	[−0.17, 0.18]	[−0.21, 0.14]	–	0.01	**0.52**	0.12
LOT	[−0.24, 0.10]	[−**0.34,** −**0.01]**	[−0.32, 0.01]	[−0.17, 0.17]	–	−0.05	−0.01
FSAS	**[0.20, 0.51]**	[−0.03, 0.31]	[−0.08, 0.27]	**[0.38, 0.64]**	[−0.26, 0.09]	–	**0.24**
No. of Absences	[−0.06, 0.15]	[−0.23, 0.18]	[−0.10, 0.31]	[−0.09, 0.32]	[−0.21, 0.20]	**[0.03, 0.42]**	–

*AIS* Athens Insomnia Scale, *CIRENS* Circadian Energy Scale, *FSAS* Feeling of School-Avoidance Scale, *LOT* Loneliness Orientation Test, *SDI* Sleep Debt Index, *TILS* Three-Item Loneliness Scale

95% confidence intervals are shown on the diagonal of the correlation coefficients. Significant coefficients are shown in bold

### Impact of sleep problems on loneliness, feelings of school avoidance, and absences

The GLM results showed that only the AIS (*β*=0.12,* p*<0.01) was related to the TILS (Nagelkerke's *R*^*2*^=0.12). The TILS (*β*=0.13,* p*<0.01) and AIS (*β*=0.05,* p*=0.07) were associated with the FSAS (Nagelkerke's *R*^*2*^=0.29), and the FSAS (*β*=0.32,* p*<0.001), SDI (*β*=−0.11,* p*=0.09), and CIRENS (*β*=0.13,* p*<0.05) were related to the number of absences (Nagelkerke's *R*^*2*^*=*0.38; Table 3).

**Table 3:** The results of general linear model

	TILS		FSAS		No. of Absences	
	*B* [SE], *β*	*p*-value	*B* [SE], *β*	*p*-value	*B* [SE], *β*	*p*-value
AIS	0.03 [0.01], 0.12	<0.001	0.01 [0.01], 0.05	0.07	0.02 [0.02], 0.10	0.15
SDI	−0.02 [0.02], −0.03	0.36	0.02 [0.02], 0.02	0.39	−0.09 [0.05], −0.11	0.09
CIRENS	−0.03 [0.02], −0.05	0.14	0.01 [0.02], 0.01	0.76	0.09 [0.04], 0.13	<0.05
LOT	−0.01 [0.03], 0.01	0.82	−0.02 [0.02], −0.01	0.52	0.03 [0.06], 0.03	0.60
TILS			0.08 [0.02], 0.13	<0.001	−0.03 [0.04], −0.05	0.44
FSAS					0.09 [0.02], 0.32	<0.001
	*R*^2^=0.12		*R*^2^=0.29		*R*^2^=0.38	

*AIS* Athens Insomnia Scale, *CIRENS* Circadian Energy Scale, *FSAS* Feeling of School-Avoidance Scale, *LOT* Loneliness Orientation Test, *SDI* Sleep Debt Index, *SE* Standardized error, *TILS* Three-Item Loneliness Scale

Values of *R*^2^ indicated the Nagelkerke's *R*^2^

### CA of loneliness and comparison of the scales between the clusters

**Fig. 1:**
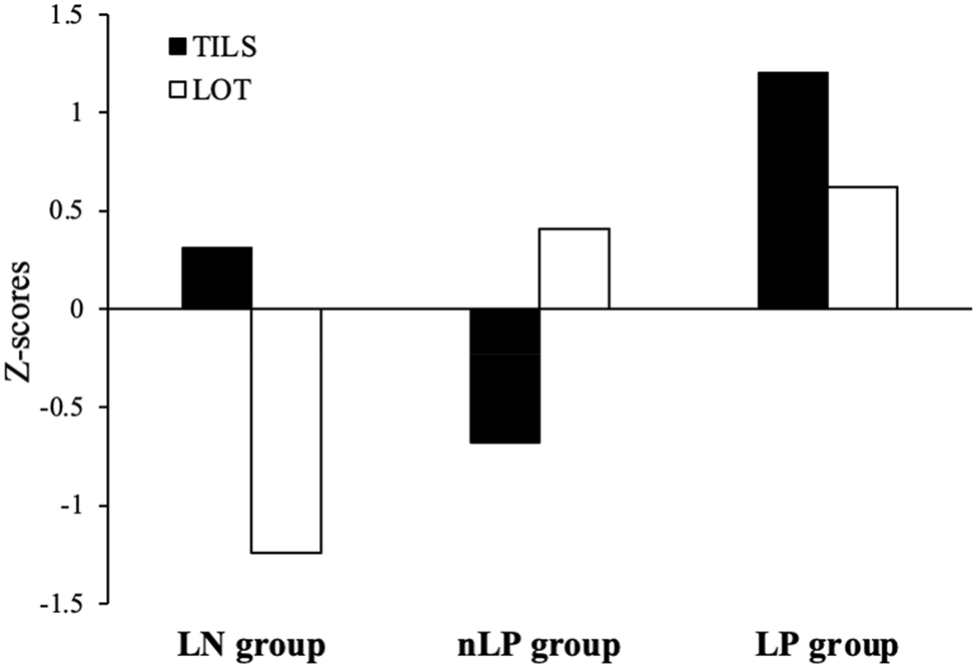
Results of the hierarchical cluster analysis. *LN* loneliness-negative orientation, *LOT* Loneliness Orientation Test, *LP* loneliness-positive orientation, *nLP* non-loneliness-positive orientation, *TILS* Three-item Loneliness Scale

The results of the hierarchical CA with ward method using the “NbClust” calculated that three clusters were optimal. Additionally, we calculated the methods of within sum of square (optimum number =3), gap statistic (optimum number =3), and silhouette (optimum number =3 or 4). We visualized 3- and 4-cluster from the results of the silhouette analysis (Supplementary 1). Shape of silhouette and the proportions of participants in each cluster were taken into account, three clusters were ultimately concluded to be relatively acceptable (Fig. 1): the first cluster was named the loneliness-negative orientation group (LN; *n*=36, 28%), the second cluster was named the non-loneliness-positive orientation group (nLP; *n*=64, 50%), and the third cluster was named the loneliness-positive orientation group (LP; *n*=28, 22%).

Since the findings of previous studies have shown that loneliness is associated with negative factors (e.g., suicide), we conducted a GLM of the LN group (with negative factors) as the comparison group. Significant differences were found in the AIS (LN vs. nLP: estimate [SE]=−1.38 [0.81]; *p*=0.09), SDI (LN vs. nLP: −0.60 [0.27]; *p*<0.05, LN vs. LP: −0.62 [0.32]; *p*=0.06), CIRENS (LN vs. LP: −0.68 [0.35]; *p*<0.05), TILS (LN vs. nLP:−1.56 [0.22], LN vs. LP: 1.24 [0.26]; *p*<0.01), LOT (LN vs. nLP: 1.55 [0.13], LN vs. LP: 1.75 [0.15]; *p*<0.01), and FSAS (LN vs. nLP: −2.25 [0.76]; *p*<0.01).

The results of multiple comparisons showed that SDI scores were higher in the LN than nLP (*g*=0.44; *p*<0.05) and LP group (*g*=0.45; *p*=0.09); CIRENS scores were higher in the LN than LP group (*g*=0.40; *p*=0.08); TILS scores were higher in the LN than nLP group (Hedges’ *g*=1.68; *p*<0.001), and lower in the LN than LP group (g=−0.99; *p*<0.001); LOT scores were lower in the LN than nLP (*g*=−2.58; *p*<0.001) and LP group (*g*=−2.51; *p*<0.001); and FSAS scores were higher in the LN than nLP group (*g*=0.59; *p*<0.01) (Table 4). There were no differences in AIS scores between the groups.

**Table 4:** Descriptive statistics in loneliness types

	Overall, mean [SE]	Loneliness types, mean [SE]			Hedges’ g [95 % CI]	
		LN	nLP	LP	LN vs. nLP	LN vs. LP
AIS	5.31 [0.35]	5.92 [0.76]	4.55 [0.38]	6.29 [0.86]	0.37 [−0.04, 0.78]	−0.08 [−0.57, 0.41]
SDI	1.57 [0.12]	2.00 [0.27]	1.41 [0.14]^a^	1.36 [0.21]^a^	**0.44 [0.03, 0.85]**	0.45 [−0.04, 0.95]
CIRENS	1.40 [0.12]	1.72 [0.25]	1.36 [0.14]	1.07 [0.32]^a^	0.28 [−0.13, 0.68]	0.40 [−0.09, 0.90]
TILS	4.34 [0.14]	4.83 [0.24]	3.27 [0.06]^a^	6.18 [0.24]^a^	**1.68 [1.21, 2.15]**	**−0.99 [−1.51, −0.47]**
LOT	−0.61 [0.08]	−1.77 [0.12]	−0.21 [0.07]^a^	−0.02 [0.12]^a^	**−2.58 [−3.12, −2.04]**	**−2.51 [−3.16, −1.86]**
FSAS	13.78 [0.34]	14.69 [0.70]	12.52 [0.41]^a^	15.50 [0.69]	**0.59 [0.17, 1.00]**	−0.20 [−0.29, 0.69]
No. of Absences	3.26 [0.48]	3.58 [0.91]	2.97 [0.59]	3.41 [1.23]	0.15 [−0.26, 0.55]	0.03 [−0.53, 0.59]

*AIS* Athens Insomnia Scale, *CIRENS* Circadian Energy Scale, *FSAS* Feeling of School-Avoidance Scale, *LN* loneliness-negative orientation, *LOT* Loneliness Orientation Test, *LP* loneliness-positive orientation, *nLP* non-loneliness-positive orientation, *SDI* Sleep Debt Index, SE Standardized error, *TILS* Three-Item Loneliness Scale

Superscripts (a) indicate significant differences with LN group

## Discussion

In this study, we aimed to develop a loneliness orientation scale to examine the relationship between feelings of loneliness and loneliness orientation; the differential impact of sleep problems on loneliness, feelings of school refusal, and the number of absences; and the relationship between the combined types of feelings of loneliness with loneliness orientation, sleep problems, and school refusal. These findings showed that feelings of school avoidance and the number of absences were associated with different sleep problems and that lonely individuals with a positive loneliness orientation did not have worse sleep conditions than those with a negative loneliness orientation.

### Factor structure of the LOT

It was revealed that the LOT consists of one factor and has high internal consistency. Additionally, the LOT was not associated with loneliness intensity (TILS), and loneliness orientation-related variables differed from loneliness intensity-related variables; loneliness intensity was associated with insomnia symptoms and feelings of school avoidance, while loneliness orientation was associated with sleep debt. Simon and Walker [25] showed that sleep deprivation may causally trigger loneliness feelings. In light of the findings of the present study, it is possible that an exacerbated effect of sleep loss is associated with negative loneliness orientation, but not the intensity of loneliness feelings. Further research is desirable.

Although many negative aspects of loneliness have been examined [6, 7], the positive aspects have not been sufficiently investigated. Therefore, the LOT measuring loneliness orientation may be a meaningful scale for examining both aspects of loneliness.

The present study is the first to introduce the concept of loneliness orientation into loneliness research. Therefore, although the factorial validity of the LOT was confirmed, concurrent validity and others were not examined. In the future, it may be useful to examine the relationship between the LOT and similar concept such as resilience, or potential attitudes towards loneliness.

### Impact of sleep problems on loneliness, feelings of school avoidance, and absences

Regression analysis with the GLM showed that insomnia and feelings of loneliness were associated with feelings of school avoidance. These findings are consistent with those of a previous study [9]. Feelings of school avoidance, sleep debt, and chronotype were related to the number of school absences. Remarkably, different types of sleep problems affect emotional aggravation and the number of school absences, although insomnia, chronotype, and sleep debt are associated with school refusal [26–28]. Additionally, increasing feelings of school avoidance may be a predictive factor for school refusal. Overall, sleep debt and chronotype had a direct effect, whereas insomnia had an indirect effect via the emotional aggravation of school refusal. Given these findings, a comprehensive assessment of sleep problems (insomnia, sleep debt, and chronotype) in adolescents is important to prevent school refusal. On the other hand, the independent variables in this study can explain about 30 to 40% of the feelings of school avoidance and the number of absences, whereas they can explain only approximately 10% for loneliness intensity. Furthermore, the impact of insomnia on the feelings of school avoidance was relatively weak. This may be because the survey covered students who were able to attend school, or because of the attribution of the influence of other variables and measurement error. Therefore, the relationship between insomnia and loneliness intensity should be interpreted with caution.

### CA of loneliness and comparison of the scales between the clusters

The results of the CA showed that almost half of the adolescents felt lonely; however, one-fourth of young people viewed loneliness in a positive manner. In particular, sleep debt and evening chronotypes were more pronounced in the LN group than in the LP group. This suggests that sleep problems may become more evident when individuals have a strong negative orientation toward loneliness, even if they experience high feelings of loneliness. By contrast, even when feelings of loneliness were high, individuals with a positive orientation toward loneliness were likely to experience fewer sleep disruptions. Given that sleep problems have been reported to increase the risk of suicide [7], these findings are in line with those of a previous study showing that lonely people with high positive affect have a lower risk of mortality than those with low positive affect [8]. The findings may be data that reinforce the RAM model [12]. This is, it is assumed that individuals with a positive orientation of loneliness overcome loneliness status because RAM is functioning normally, while those with negative orientation of loneliness prolong their loneliness status because of RAM malfunction. There is no previous research on this point, and it is still a matter of speculation. Further research is desirable.

On the other hands, there were no significant differences in feelings of school refusal or the number of absences between the LN and LP groups. Therefore, although loneliness intensity affects feelings of school refusal, the positive aspect of overcoming loneliness may contribute to other factors. For example, a qualitative study of older adults in nursing homes found that they coped by reframing their experiences and identifying new meanings in their lives [29]. That is, the positive aspect of overcoming loneliness may be related to self-growth and well-being. In addition, high intensity of loneliness was associated with low well-being and low self-esteem in Nordic adolescents [30]. In light of the findings of this study, therefore, the relationship between the positive aspects of overcoming loneliness, such as loneliness orientation and self-fulfillment factors—such as self-growth, resilience, and well-being—needs further investigation.

We have some limitations as follows: First, a sample size was relatively small, although more participants were recruited than the pre-calculated sample size; second, the sample was limited to high school students; third, only students who were able to attend school were included; and finally, the causal relationship between sleep and loneliness intensity/orientation and the confounders between these factors is not clear. To examine the relationship between sleep, loneliness intensity/orientation, and school refusal in adolescents, it is necessary to conduct future studies covering a wide range of students, from junior high school to university students. It may also be necessary to examine the relationship between sleep problems and loneliness intensity/orientation among students who are not currently attending school.

In summary, different sleep problems were revealed to be involved in feelings of school refusal and absenteeism; even when loneliness intensity is strong, outcomes such as sleep problems vary depending on the individual's orientation toward loneliness in adolescents. Future research should explore the factors related to loneliness orientation and self-fulfillment and examine the effects of loneliness intensity and orientation in other age groups.

## Supplementary Information

Below is the link to the electronic supplementary material.

Supplementary file1 (PPTX 178 KB)

## Data Availability

The data that support the findings of this study are openly available in Zenodo at 10.5281/zenodo.14166866.
