# Supplementary material for: How is loneliness orientation implicated in the relationship between sleep problems, loneliness intensity, and school refusal in adolescents?
Source: Sleep Biol Rhythms. 2025 Apr 24;23(4):419–26. doi: 10.1007/s41105-025-00586-9 (PMC12450842; doi:10.1007/s41105-025-00586-9)
Supplement: Supplementary file 1 — Supplementary file1 (PPTX 178 KB) [file 41105_2025_586_MOESM1_ESM.pptx]

## Slide 1
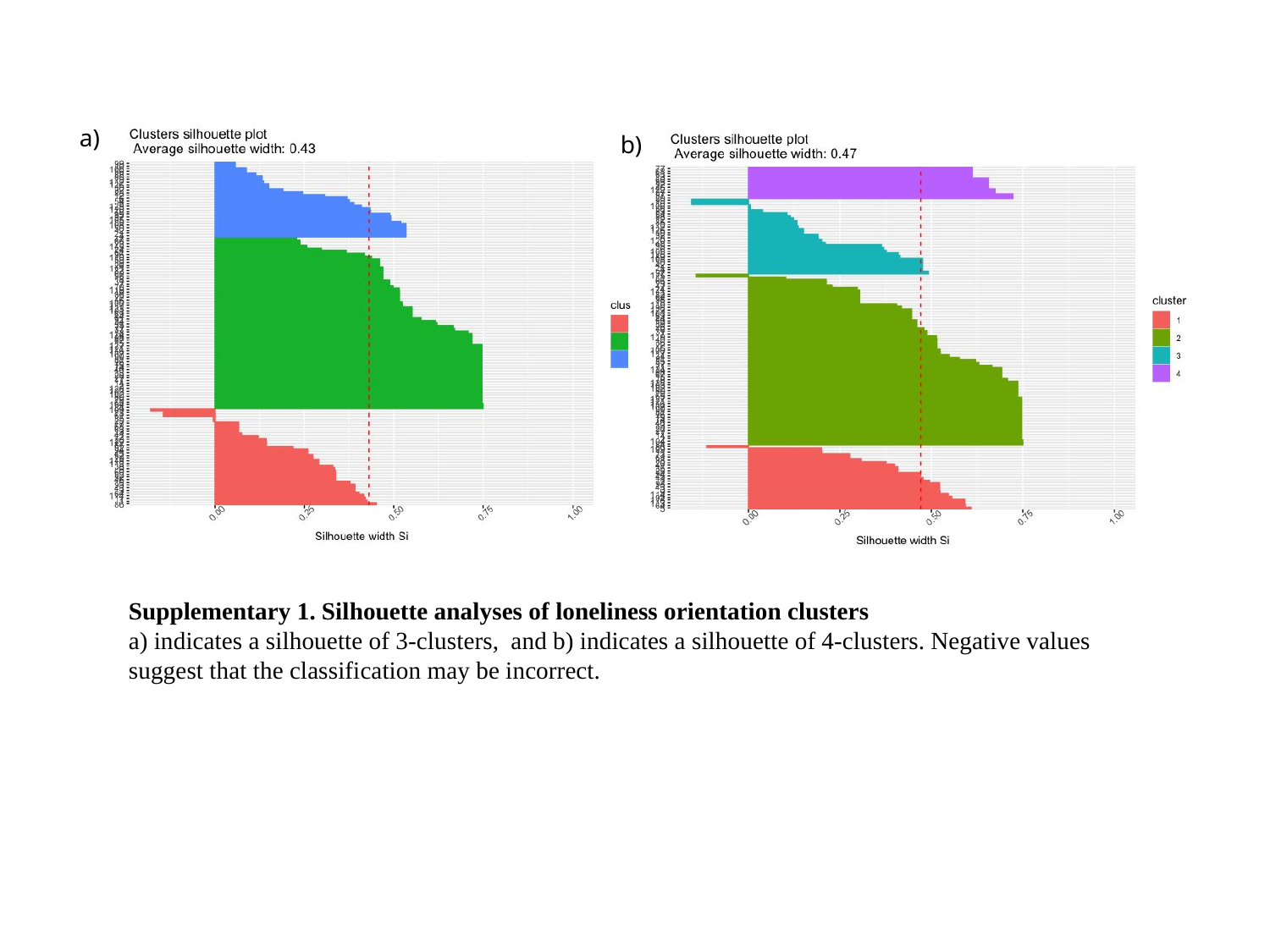

a)
b)
Supplementary 1. Silhouette analyses of loneliness orientation clusters
a) indicates a silhouette of 3-clusters, and b) indicates a silhouette of 4-clusters. Negative values suggest that the classification may be incorrect.
